# Prevalence and predictive factors for bilateral carpal tunnel syndrome by electrodiagnosis: A retrospective study

**DOI:** 10.1371/journal.pone.0260578

**Published:** 2021-12-23

**Authors:** Apiradee Singjam, Kanchana Charoentanyarak, Jittima Saengsuwan

**Affiliations:** 1 Rehabilitation Medicine Unit, Khon Kaen Hospital, Khon Kaen, Thailand; 2 Department of Rehabilitation Medicine, Faculty of Medicine, Khon Kaen University, Khon Kaen, Thailand; Universita degli Studi di Firenze, ITALY

## Abstract

**Introduction:**

Carpal tunnel syndrome (CTS) is the most common compressive neuropathy. Patients who have unilateral symptoms are frequently found to have bilateral CTS by electrodiagnostic (EDx) study. We aimed to (a) study the prevalence and identify the predictive factors for bilateral CTS diagnosed by EDx; and (b) develop a model to predict bilateral CTS.

**Methods:**

The retrospective clinical and EDx data of patients with CTS were collected and analyzed using the Chi-squared test and multiple logistic regression analysis. A model was fitted, and the best cutoff point determined. Calibration and discrimination performance of the model were performed.

**Results:**

A total of 327 patients with a mean age of 50.0 years were enrolled. Most were women (82.6%), and the most common presenting symptom was hand numbness (93.6%). The median duration of symptoms was 60 days. The prevalence of bilateral CTS was 80.7%. In the multivariate analysis, the predictive factors for bilateral CTS were the presence of bilateral symptoms (AOR 6.7 [95%CI 3.1–14.3]), thenar muscle weakness (AOR 3.9 [95%CI 1.3–11.6]), and age ≥ 45 years (AOR 2.5 [95%CI 1.3–4.6]). The logistic regression model was fitted, and the best cutoff point determined. The area under the receiver operating curve (AUC) was 0.76. The respective optimism-corrected C index and Somers’ D was 0.762 and 0.524.

**Conclusion:**

The prevalence of bilateral CTS was 80.7%. Our findings suggest bilateral CTS was predicted with adequate diagnostic accuracy by bilateral symptoms, age ≥ 45 years, and thenar muscle weakness.

## Introduction

Carpal tunnel syndrome (CTS) is a clinical syndrome characterized by numbness, tingling, burning, and/or pain associated with localized compression of the median nerve at the wrist [1]. CTS is the most common cause of compressive neuropathy [2]. Presenting symptoms usually include numbness, paresthesia, and pain in the area of the cutaneous supply of the median nerve [3, 4]. Some patients report weakness and atrophy of the thenar area, interfering with hand function.

The diagnostic yield of CTS based on a general physical examination is poor [5–8], so electrodiagnostic (EDx) studies have been adopted because of their greater sensitivity and specificity [2], particularly before treatment or surgical intervention [9].

Although presenting symptoms may be unilateral, CTS is commonly found bilaterally. Previous studies reported the prevalence of bilateral CTS between 59 and 87% [10–13]. The factors associated with bilateral CTS are age and BMI [14]. Approximately half the patients who presented with unilateral symptoms were found to have bilateral CTS by electrodiagnostic study. There is an increased risk of symptomatic CTS in patients with an abnormal neurophysiologic study (i.e., prolonged median sensory latency without symptoms over 6 years) [15]. Most patients with unilateral symptomatic CTS but bilateral CTS (diagnosed by EDx) later present bilateral symptoms [10]. We aimed to determine the prevalence of bilateral CTS and to identify predictive factors for bilateral CTS. We also sought to explore whether these factors could be implemented as a predictive tool for bilateral CTS.

## Materials and methods

A retrospective study was conducted in Khon Kaen Hospital, Thailand, between March 2020 and March 2021. The study was reviewed and approved by the Khon Kaen Hospital Ethics Committee for Human Research (KEXP63009), performed according to the ethical principles described in the Declaration of Helsinki and all methods were performed in accordance with the relevant guidelines and regulations. Informed consent was waived for this study. The study population included patients who underwent electrodiagnostic study between January 2012 and December 2018. Our inclusion criteria included (a) was ≥ 18 years age, (b) underwent electrodiagnostic study on both hands, and (c) was diagnosed with CTS by EDx in at least one hand. Exclusion criteria were polyneuropathy or concomitant cervical radiculopathy. We excluded patients who had undergone treatment with steroid injection or carpal tunnel decompression before the EDx study.

We reviewed the medical records of the CTS patients. EDx studies were performed by board-certified rehabilitation physicians using the Nicolet Biomedical Viking quest system (Nicolet Biomedical, Madison, WI, USA). The diagnostic criteria and severity of CTS were graded according to the American Association of Neuromuscular and Electrodiagnostic Medicine AANEM: (1) mild CTS is defined as prolonged sensory latency (>3.5 msec) with normal motor nerve conduction study (NCS); (2) moderate CTS is defined as abnormal median sensory (>3.5 msec) and motor latency (>4.2 msec); and, (3) severe CTS is defined as the abnormalities mentioned above with evidence of axonal loss defined by low amplitude or absent sensory nerve action potential (SNAP), low amplitude or absence of thenar compound muscle action potential (CMAP) or needle EMG showing signs of membrane instability [1, 16].

### Statistical analysis

The sample size calculation for model development for binary outcomes requires there be more than 20 events per candidate predictor parameter (EPP) to prevent overfitting [17]. Chi-squared and Fisher’s Exact test were performed to determine factors associated with bilateral CTS. The variables included in this study were sex, age (≥45 years), obesity (BMI≥30 kg/m^2^) [18–20], diabetes, thyroid disease, connective tissue disorders, thenar weakness, thenar atrophy, bilateral complaints, duration of symptoms (≥90 days). The cut-off point for the duration of symptoms was calculated using Youden’s index [21]. When the p-value was < 0.2, these variables were included in the logistic regression analysis to determine the predictive factors for bilateral CTS. The predictive model was constructed using a stepwise selection method [22] then the model was fitted, and the risk score and probability of having bilateral CTS in each individual calculated. The best cutoff point was the change in the risk score that most effectively separated patients with and without bilateral CTS within in the Receiver Operator Characteristic (ROC) curve using Youden’s index [21]. The area under the ROC curve was defined as excellent (≥ 0.90); adequate (0.70–0.89); or, poor (< 0.70) [23]. A P < 0.05 was considered statistically significant. The data were resampled using the bootstrapping technique of 1000 iterations to evaluate the internal validity of the model [24]. The optimism-corrected discrimination index of the model was evaluated using the C-statistic and the optimism-corrected calibration index was expressed as the Somers’ D rank correlation, whereby D = 2C-1 [25]. A C-index of 0.5 indicated no discrimination, and a value of 1.0 indicated perfect discrimination of patients with different outcomes [25]. A higher Somers’ D indicated a better performance of the model. Statistical analyses were performed using Stata version 13.1 (Stata Statistical Software: Release 13. College Station, TX: StataCorp LP.).

## Results

A total of 327 patients (270 women, 57 men), mean age 50 ± 11 years, met the inclusion criteria. Most (56.3%) patients complained of unilateral hand problems. The three most common complaints were hand numbness (93.6%), pain (58.1%), and weakness (16.5%). The median duration of symptoms was 60 days. Nearly one-third of patients had an underlying disease: hypertension (17.1%), thyroid disease (2.8%), and/or diabetes mellitus (5.2%) (Table 1).

**Table 1:** Demographic data (n = 327).

Variables	n (%)
**Age** (yrs), mean (SD)	50.0 (11.0)
**Sex**, n (%)	
Men	57 (17.4%)
Women	270 (82.6%)
**Underlying**, n (%)	88 (26.9%)
Hypertension	56 (17.1%)
Diabetes	17 (5.2%)
Thyroid disease	9 (2.8%)
**Duration of symptoms** (days), median (IQR)	60 (30–180)
**Side of symptoms**, n (%)	
Right	72 (22.0%)
Left	112 (34.3%)
Bilateral	143 (43.7%)
**Presenting symptoms**, n (%)	
Numbness	306 (93.6%)
Pain	190 (58.1%)
Weakness	54 (16.5%)
Atrophy	23 (7.0%)

Most patients (n = 264, 80.7%) had EDx diagnosed bilateral CTS. Most (93%) patients with initial complaints of bilateral symptoms had bilateral CTS. In patients presenting with unilateral symptoms, more than two-thirds (71.2%) were diagnosed with bilateral CTS. When comparing the EDx abnormality in patients with unilateral symptoms who had bilateral CTS, only half of the symptomatic hands had more severe CTS (51.9%), and nearly half presented a similar EDx severity. Only 2.3% of CTS symptomatic hands were less severe on the asymptomatic side (Table 2).

**Table 2:** Nerve conduction study results in 327 patients.

Variables	N (%)
**Unilateral abnormality**	63 (19.3%)
Mild	32 (50.8%)
Moderate	21 (33.3%)
Severe	10 (15.9%)
**Bilateral abnormality**	264 (80.7%)
**Right**	
Mild	63 (23.9%)
Moderate	141 (53.4%)
Severe	60 (22.7%)
**Left**	
Mild	75 (28.4%)
Moderate	137 (51.9%)
Severe	52 (19.7%)

**Fig 1:**
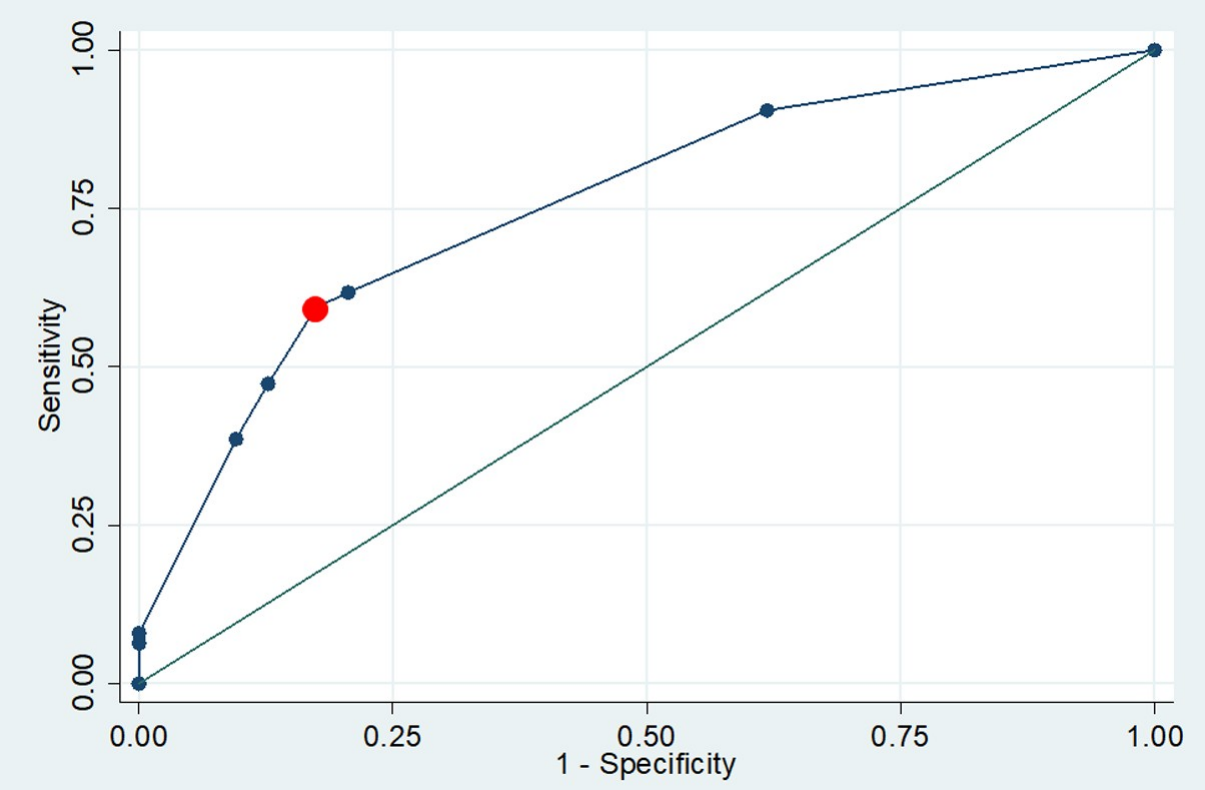
ROC curves–plot of sensitivity versus 1-specificity values for the model: Bilateral CTS score = 0.1 + (1.4 x thenar muscle weakness) + (1.9 x bilateral symptoms) + (0.9 x AGE ≥ 45 yrs). Scoring: thenar muscle weakness: no = 0, yes = 1; Bilateral symptoms: no = 0, yes = 1; and age ≥45 years: no = 0, yes = 1. The best cutoff score was 2.0.

Factors associated with bilateral CTS were bilateral symptoms, BMI ≥ 30 kg/m^2^, age ≥ 45 years, duration of symptoms ≥ 90 days, the presence of thenar muscle weakness, and thenar muscle atrophy. Sex and diabetes mellitus were not found associated with bilateral CTS (Table 3). In the multivariate analysis, predictive factors for bilateral CTS included the presence of bilateral symptoms (AOR 6.7 [95% CI 3.1–14.3]), thenar muscle weakness (AOR 3.9 [95% CI 1.3–11.6]), and age ≥ 45 years (AOR 2.5 [95% CI 1.3–4.6]) (Table 4). According to the logistic regression, the bilateral CTS score = 0.1 + (1.4 x thenar muscle weakness) + (1.9 x bilateral symptoms) + (0.9 x AGE ≥ 45 years). Scoring: thenar muscle weakness: no = 0, yes = 1; Bilateral symptoms: no = 0, yes = 1; and age ≥45 years: no = 0, yes = 1. The best cutoff score was 2.0, which had a sensitivity of 59.5%, a specificity of 82.5%, a positive likelihood ratio of 3.4, and a negative likelihood ratio of 0.5. The area under the ROC curve (AUC) was 0.76 (95% CI 0.70–0.82) (Fig 1). After resampling, the respective 1000-replication bootstrap yielded an optimism-corrected C index, and Somers’ D was 0.762 and 0.524.

**Table 3:** Factors associated with bilateral CTS.

Variables (n)	Unilateral, n (%)	Bilateral, n (%)	P-value
	(n = 63)	(n = 264)	
Age<45 years (96)	29 (30.2)	67 (69.8)	0.001
Age≥45 years (231)	34 (14.7)	197 (85.3)	
Women (270)	50 (18.5)	220 (81.5)	0.56
Men (57)	13 (22.8)	44 (77.2)	
BMI < 30 kg/m^2^ (177)*	59 (21.1)	221 (78.9)	0.029
BMI ≥ 30 kg/m^2^ (150)	4 (8.5)	43 (91.5)	
No DM (310)*	61 (19.7)	249 (80.3)	0.54
DM (17)	2 (11.8)	15 (88.2)	
No thyroid disease (318)*	59 (18.6%)	259 (81.4%)	0.067
Thyroid disease (9)	4 (44.4%)	5 (55.6%)	
No connective tissue disease (320)*	61 (19.1%)	259 (80.9%)	0.62
Connective tissue disease (7)	2 (28.6%)	5 (71.4%)	
Duration <90 days (168)	40 (23.8)	128 (76.2)	0.034
Duration ≥90 days (159)	23 (14.5)	136 (85.5)	
Unilateral symptoms (184)	54 (29.4)	130 (70.7)	<0.001
Bilateral symptoms (143)	9 (6.3)	134 (93.7)	
No thenar muscle weakness (273)*	59 (21.6)	214 (78.4)	0.022
Thenar muscle weakness (54)	4 (7.4)	50 (92.6)	
No thenar muscle atrophy (304)*	62 (20.4)	242 (79.6)	0.060
Thenar muscle atrophy (23)	1 (4.3)	22 (95.7)	

*Fisher’s exact test.

**Table 4:** Unadjusted and adjusted odds ratio between different variable and bilateral CTS.

	Unadjusted OR (95%CI)	P value	Adjusted OR (95%CI)	P value
Unilateral symptom	1		1	
Bilateral symptoms	6.2 (2.9–13.0)	<0.001	6.7 (3.1–14.3)	<0.001
No thenar muscle weakness	1		1	
Thenar muscle weakness	3.4 (1.2–9.9)	0.022	3.9 (1.3–11.6)	0.015
Age < 45 years	1		1	
Age ≥ 45 years	2.5 (1.4–4.4)	0.002	2.5 (1.3–4.6)	0.003
BMI < 30 kg/m^2^	1			
BMI ≥ 30 kg/m^2^	2.9 (1.0–8.3)	0.029	NS	NS
Duration <90 days	1			
Duration ≥90 days	1.9 (1.1–3.3)	0.034	NS	NS
No thenar muscle atrophy	1			
Thenar muscle atrophy	5.6 (0.7–42.6)	0.094	NS	NS
No thyroid disease	1			
Thyroid disease	0.3 (0.1–1.1)	0.067	NS	NS

Abbreviations: OR = Odds ratio, NS = not significant.

## Discussion

This study aimed to determine the prevalence of bilateral CTS and factors associated with CTS. The prevalence of bilateral CTS was 80.7%. The prevalence of bilateral CTS was in line with previous studies that documented 59–87% [10–13]. In patients with only unilateral symptoms, bilateral CTS was found in 71.2%.

The present study identified bilateral symptoms, thenar muscle weakness, age ≥ 45 years, duration of symptoms ≥ 90 days, BMI ≥30 kg/m^2^, and thenar muscle atrophy as associated with bilateral CTS, which is consistent with previous studies. Kouyoumdjian et al. showed that age and severity of CTS are factors associated with bilateral CTS [14]. We also found that longer duration of symptoms was an independent factor associated with bilateral CTS; however, our duration (90 days) was shorter than previous studies. For example, Lewańska et al. found that bilateral CTS had a longer duration of symptoms (i.e., 4.01 years in bilateral CTS and 1.7 years in unilateral CTS) [13]. Similarly, Bagatur et al. reported that the duration of symptoms was longer in bilateral CTS (3 years) than unilateral CTS (1 year). Padua et al. confirmed that most patients with unilateral symptomatic CTS, but bilateral CTS diagnosed by EDx, showed bilateral symptoms later [10]. This finding is consistent with Bagatur et al. who also found that among patients with unilateral symptoms, 76% developed symptoms in the non-operated hand in a mean interval of 3.2 years [11]. Bagatur et al. pointed out that bilaterality may be time-dependent, and clinicians should inform patients with unilateral CTS about the risk of developing CTS in the contralateral hand [11, 13].

Although CTS was more common in women [26], we did not find any association between sex and bilateral CTS, i.e., women did not show a higher proportion of bilateral CTS than unilateral CTS. Diabetes mellitus (DM) was not found to be associated with bilateral CTS. Interestingly, recent epidemiological studies also found no significant association between DM and CTS [27].

The multivariate analysis revealed that patients with bilateral symptoms, thenar muscle weakness, and age ≥ 45 years had a greater probability of having bilateral CTS (OR 6.7, 3.9, and 2.5, respectively). We developed an algorithm for estimating the probability of having bilateral CTS. The diagnostic accuracy of the model was considered adequate (AUC 0.76). The respective optimism-corrected C index and Somers’ D was 0.762 and 0.524, indicating moderate accuracy.

The study has some limitations because the research was retrospective, so relevant data regarding occupations requiring repetitive tasks, previous wrist trauma or handedness were incomplete. We excluded patients who had previous interventional treatment for CTS or did not have electrodiagnostic study on both hands. This exclusion may limit the generalizability of the study. The diagnosis of CTS in an asymptomatic hand was based on the sensory and motor conduction studies across the wrist of the median nerve. In such cases, we did not perform a further combined sensory index (CSI). CSI is a summary score of three sensory latency differences: (a) transpalmar latency differences (palmdiff), (b) the median ulnar comparison with the ring finger (ringdiff), and (c) the median radial comparison with the thumb (thumbdiff), providing greater diagnostic sensitivity and specificity of CTS [28]. This may result in an underestimation of the prevalence of bilateral CTS. Some variables such as age, BMI, and duration of carpal tunnel syndrome were dichotomized which may reduce statistical power and information [29]. The model developed in our study has not been externally validated thus the generalizability of the results may be limited. Future studies should be prospective, and all variables related to CTS and bilateral CTS development should be included. In case of negative findings from routine EDx study, CSI should be considered. Long-term follow-up should be done to determine the significance of the findings of bilateral CTS.

## Conclusion

The prevalence of bilateral CTS was 80.7%. Our findings suggest bilateral symptoms, age ≥ 45 years, and thenar muscle weakness can predict bilateral CTS with adequate diagnostic accuracy.

## Supporting information

S1 File Dataset. (XLSX)

## List of abbreviations

AOR: adjusted odds ratio
AUC: Area under the receiver operating characteristic curve
CTS: carpal tunnel syndrome
CMAP: compound muscle action potential
EDx: electrodiagnosis
EMG: Electromyography
NCS: nerve conduction study
OR: odds ratio
